# Video-assisted thoracoscopic lobectomy is feasible for selected patients with clinical N2 non-small cell lung cancer

**DOI:** 10.1038/s41598-020-72272-4

**Published:** 2020-09-16

**Authors:** Jae Kwang Yun, Geun Dong Lee, Sehoon Choi, Hyeong Ryul Kim, Yong-Hee Kim, Seung-Il Park, Dong Kwan Kim

**Affiliations:** Department of Thoracic and Cardiovascular Surgery, Asan Medical Center, University of Ulsan College of Medicine, 88, Olympic-ro 43-gil, Songpa-gu, Seoul, 05505 Republic of Korea

**Keywords:** Surgical oncology, Non-small-cell lung cancer

## Abstract

Few studies have evaluated the usefulness of video-assisted thoracoscopic surgery (VATS) for advanced-stage lung cancer. We aimed to evaluate the feasibility of VATS for treating clinical N2 (cN2) lung cancer. A retrospective cohort analysis was performed with data from 268 patients who underwent lobectomy for cN2 disease from 2007 to 2016. Using propensity score-based inverse probability of treatment weighting (IPTW), perioperative and long-term survival outcomes were compared. We performed VATS and open thoracotomy on 121 and 147 patients, respectively. Overall, VATS was preferred for patients with peripherally located tumors (*p* < 0.001). After IPTW-adjustment, all preoperative information became similar between the groups. Compared to thoracotomy, VATS was associated with shorter hospitalization (7.7 days vs. 9.1 days, *p* = 0.028), despite equivalent complete resection rates (92.6% vs. 90.5%, *p* = 0.488) and dissected lymph nodes (mean, 31.9 vs. 29.4, *p* = 0.100). On IPTW-adjusted analysis, overall survival (50.5% vs. 48.4%, *p* = 0.127) and recurrence-free survival (60.5% vs 44.6%, *p* = 0.069) at 5 years were also similar between the groups. Among selected patients with resectable cN2 disease and peripherally located tumors, VATS is feasible, associated with shorter hospitalization and comparable perioperative and long-term survival outcomes, compared with open thoracotomy.

## Introduction

Since the introduction of video-assisted thoracoscopic surgery (VATS) for pulmonary lobectomy in 1994^1^, it has become a preferred surgical approach for early-stage non-small cell lung cancer (NSCLC)^2,3^. Compared with open thoracotomy, VATS has several advantages, such as less incisional pain, better preservation of chest muscles and respiratory function, shortened length of hospitalization, decreased postoperative morbidity, and enhanced compliance with adjuvant chemotherapy^4–7^. However, few studies have evaluated the usefulness of VATS for treating advanced-stage NSCLC^8,9^, and most such studies have been investigated only the treatment of early-stage disease^4–7^. Furthermore, no study has analyzed the clinical utility of VATS lobectomy for stage III (N2) NSCLC.

While the presence of N2 metastasis generally indicates systemic disease, and the standard treatment is multidisciplinary therapy based on chemotherapy, radiotherapy, or chemoradiotherapy combined with surgery^10,11^. Upfront surgery is occasionally performed on patients with resectable N2 disease, especially those with single N2 disease^12–14^. With accumulating clinical experience and technical development, the eligibility criteria for VATS lobectomy have been expanded to advanced lung cancer, including clinical N2 (cN2) disease. However, owing to the limited comparative data on VATS and open thoracotomy in this cohort, applying VATS to patients with cN2 disease raises concerns about the possibility of incomplete lymph node (LN) dissection, technical challenges for anatomical dissection, and the consequent compromised long-term oncologic outcomes. In this study, we evaluated the feasibility of VATS lobectomy for patients with clinically suspected N2 node metastasis. We also compared long-term oncologic outcomes between VATS and thoracotomy using an inverse probability of treatment weighting (IPTW) technique.

## Results

**Figure 1:**
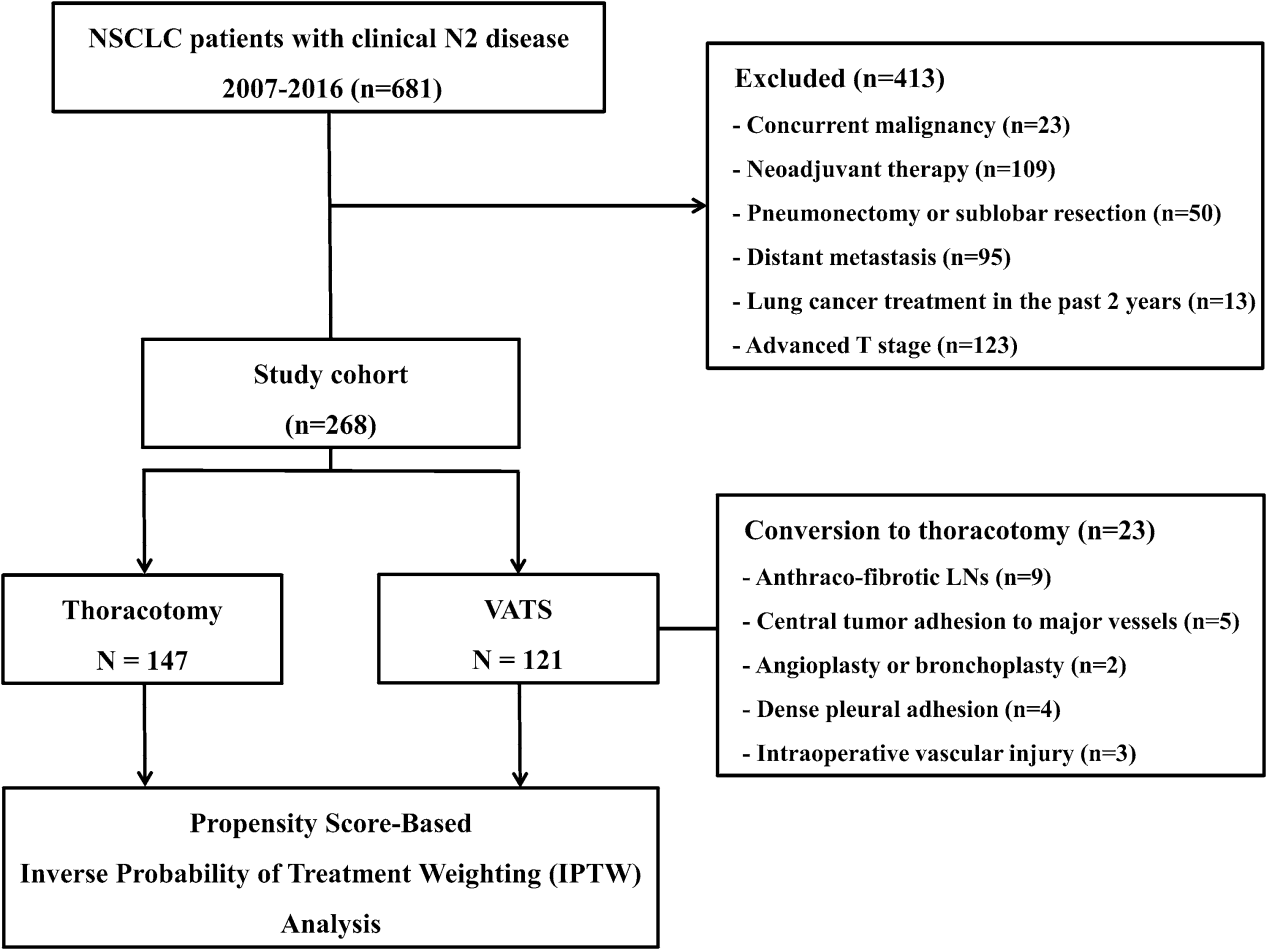
Schematic diagram of the study subject selection process (clinical T1-2, N2, M0 patients).

A total of 268 patients with cN2 disease who underwent lobectomy during the study period were enrolled. Among them, 121 (45.1%) and 147 (54.9%) patients underwent VATS and thoracotomy, respectively. Conversion to thoracotomy occurred in 23 of 121 patients (19.0%) who initially underwent VATS lobectomy, due to anthraco-fibrotic LNs (n = 9), centrally located tumor adhesion to major vessels (n = 5), angioplasty or bronchoplasty (n = 2), dense pleural adhesions (n = 4), or intraoperative vascular injury (n = 3) (Fig. 1).

### Baseline characteristics

The median follow-up duration after surgery was 43 months (interquartile range, 24–71 months). The baseline demographic and tumor characteristics are listed in Table 1. On IPTW-adjusted analysis, clinical variables, such as age, sex, comorbidities, pulmonary function, tumor location, and clinical stage, were all balanced (all standardized mean differences [SMDs] were ≤ 0.1) and showed no significant differences (all *p* values were > 0.05) between the two groups (Table 1).

**Table 1:** Clinicopathologic characteristics of patients with clinical N2 disease before and after inverse probability of treatment weighting (N = 268).

Variables	Overall cohort			IPTW-adjusted cohort			
	VATS (n = 121)	Thoracotomy (n = 147)	*p* value	VATS (n = 121)	Thoracotomy (n = 147)	*p* value	SMD
**Operation period**							
2007–2010	33 (27.3)	59 (40.1)	0.038	37 (30.6)	50 (34.0)	0.616	0.073
2011–2013	27 (22.3)	31 (21.1)	0.926	29 (24.0)	32 (21.8)	0.794	0.039
2014–2016	61 (50.4)	57 (38.8)	0.074	55 (45.4)	65 (44.2)	0.809	0.035
Age, year	64.0 ± 10.4	62.4 ± 9.5	0.189	63.8 ± 10.2	63.4 ± 9.5	0.742	0.046
Sex, male	76 (62.8)	120 (81.6)	0.001	87 (71.9)	111 (75.5)	0.582	0.081
History of smoking	76 (62.8)	119 (81.0)	0.001	88 (72.7)	111 (75.5)	0.687	0.058
**Comorbidities per patient, n**							
0	46 (38.0)	64 (43.5)	0.430	53 (43.8)	64 (43.5)	0.959	0.008
1	48 (39.7)	52 (35.4)	0.551	44 (36.4)	51 (34.7)	0.852	0.027
2	20 (16.5)	27 (18.4)	0.816	20 (16.5)	28 (19.0)	0.633	0.067
≥ 3	7 (5.8)	4 (2.7)	0.343	4 (3.3)	4 (2.7)	0.670	0.051
History of pulmonary Tbc	10 (8.3)	11 (7.5)	0.993	12 (9.9)	15 (10.2)	0.980	0.004
**Pulmonary function**							
FEV1, %	87.6 ± 14.7	84.5 ± 16.2	0.109	87.4 ± 14.9	86.7 ± 16.7	0.793	0.039
DLCO, %	82.3 ± 14.2	82.6 ± 16.9	0.858	81.9 ± 14.6	82.6 ± 17.2	0.760	0.047
**Tumor location**			< 0.001			0.546	0.090
Central	28 (23.1)	78 (53.4)		52 (43.0)	70 (47.6)		
Peripheral	93 (76.9)	68 (46.6)		69 (57.0)	77 (52.4)		
**Lobar location**							
Right upper	32 (26.4)	45 (30.6)	0.539	38 (31.4)	46 (31.3)	0.966	0.006
Right middle	9 (7.4)	9 (6.1)	0.855	7 (5.8)	11 (7.5)	0.659	0.062
Right lower	31 (25.6)	48 (32.7)	0.262	36 (29.8)	41 (27.9)	0.806	0.036
Left upper	30 (24.8)	35 (23.8)	0.965	26 (21.5)	34 (25.0)	0.739	0.046
Left lower	19 (15.7)	10 (6.8)	0.033	14 (11.5)	15 (10.1)	0.615	0.069
Clinical tumor size, mm	29.8 ± 10.1	34.4 ± 9.1	< 0.001	32.1 ± 10.1	32.7 ± 9.1	0.672	0.062
**Histology**							
ADC	77 (63.6)	49 (33.3)	< 0.001	61 (50.4)	67 (45.6)	0.522	0.095
SqCC	37 (30.6)	85 (57.8)	< 0.001	52 (43.0)	69 (46.9)	0.587	0.081
Others	7 (5.8)	12 (8.2)	0.606	8 (6.6)	11 (7.5)	0.928	0.012
**8th clinical T factor**			< 0.001			0.836	0.030
T1	46 (38.0)	20 (13.6)		30 (24.8)	34 (23.1)		
T2	75 (62.0)	127 (86.4)		91 (75.2)	113 (76.9)		

Data are presented as number (%) unless otherwise noted. *IPTW* inverse probability of treatment weighting, *VATS* video-assisted thoracoscopic surgery, *SMD* standardized mean difference, *FEV1* forced expiratory volume during the first second, *DLCO* diffusing capacity of carbon monoxide, *Tbc* tuberculosis, *ADC* adenocarcinoma, *SqCC* squamous cell carcinoma.

### Perioperative data

Table 2 presents the operative profiles of the two groups. The mean numbers of harvested LNs (31.9 vs. 29.4, *p* = 0.100) and the rates of complete resection (92.6% vs. 90.5%, *p* = 0.488) were not significantly different between the VATS and thoracotomy groups. The mean duration of hospital stay was shorter in the VATS group, regardless of IPTW adjustment (before adjustment: 7.3 days vs. 9.2 days, *p* = 0.001; after adjustment: 7.7 days vs. 9.1 days, *p* = 0.028). Additionally, the mean time to start adjuvant chemotherapy was also shorter in the VATS group (before adjustment: 36.7 days vs. 43.4 days, *p* = 0.036; after adjustment: 36.9 days vs. 43.1 days, *p* = 0.033). The rate of adjuvant chemotherapy was similar between the two groups overall (53.7% vs. 52.4%, *p* = 0.924); however, it became different after IPTW adjustment (54.5% vs. 46.9%, *p* = 0.033).

**Table 2:** Perioperative profiles of the study patients.

Variables	Overall cohort			IPTW-adjusted cohort		
	VATS (n = 121)	Thoracotomy (n = 147)	*p* value	VATS (n = 121)	Thoracotomy (n = 147)	*p* value
Hospital days, days	7.3 ± 4.7	9.2 ± 4.4	0.001	7.7 ± 4.6	9.1 ± 4.2	0.028
**Resection margins**			0.079			0.488
R0	114 (94.2)	128 (87.1)		112 (92.6)	133 (90.5)	
R1	7 (5.8)	19 (12.9)		9 (7.4)	14 (9.5)	
**Lymph node dissection**						
Lymph nodes harvested, n	31.6 ± 14.4	30.0 ± 10.0	0.634	31.9 ± 13.3	29.4 ± 9.6	0.100
Positive lymph nodes, n	4.7 ± 5.2	4.3 ± 5.9	0.316	4.6 ± 5.1	4.7 ± 5.9	0.980
Pathological tumor size	33.3 ± 12.1	36.7 ± 11.4	0.018	36.0 ± 12.1	35.3 ± 11.4	0.684
**8th pathologic T factor**			0.283			0.543
T1	35 (28.9)	31 (21.1)		25 (20.7)	40 (27.2)	
T2	71 (58.7)	92 (62.6)		77 (63.6)	86 (58.5)	
T3	15 (12.4)	24 (16.3)		19 (15.7)	21 (14.3)	
**8th pathologic N factor**			0.365			0.847
N0	30 (24.8)	42 (28.6)		31 (25.6)	37 (25.2)	
N1	8 (6.6)	16 (10.9)		8 (6.6)	13 (8.8)	
N1a	7 (5.8)	15 (10.2)		7 (5.8)	12 (8.1)	
N1b	1 (0.8)	1 (0.7)		1 (0.8)	1 (0.7)	
N2	83 (68.6)	89 (60.5)		82 (67.8)	97 (66.0)	
N2a1	18 (14.9)	13 (8.8)		14 (11.6)	15 (10.2)	
N2a2	28 (22.3)	39 (26.5)		32 (26.4)	39 (26.5)	
N2b	38 (31.4)	37 (25.2)		36 (29.8)	43 (29.3)	
**8th pathologic stage**			0.452			0.882
I	20 (16.5)	27 (18.4)		18 (14.9)	24 (16.3)	
II	17 (14.0)	28 (19.0)		20 (16.5)	22 (15.0)	
III	84 (69.4)	92 (62.6)		83 (68.6)	101 (68.7)	
**Adjuvant chemotherapy**	65 (53.7)	77 (52.4)	0.924	66 (54.5)	69 (46.9)	0.287
Time to start chemotherapy, days	36.7 ± 18.1	43.4 ± 22.1	0.036	36.9 ± 14.3	43.1 ± 22.3	0.033
Adjuvant radiotherapy	55 (45.5)	68 (46.3)	0.993	56 (46.3)	66 (44.9)	0.829

Data are presented as number (%) unless otherwise noted. *IPTW* inverse probability of treatment weighting; video-assisted thoracoscopic surgery, *R0* completely resected tumor, *R1* microscopic residual tumor.

Early mortality (30-day or in-hospital) occurred in one patient each in the VATS (0.8%) and thoracotomy (0.7%) groups (Table 3). The early death in the VATS group was caused by an aggravation of interstitial pulmonary fibrosis, and the early death in the thoracotomy group was caused by postoperative pneumonia. In terms of postoperative complications, there were no significant differences between the VATS and thoracotomy groups (Table 3, Cohort 1). When patients were divided into three groups (VATS, planned thoracotomy, and thoracotomy conversion), the rate of pneumonia was the lowest in the VATS group (3.1%), whereas it was similar between the thoracotomy (7.5%) and conversion groups (8.7%).

**Table 3:** Postoperative complications and the impact of VATS.

Variables	Cohort 1			Cohort 2			
	VATS (n = 121)	Thoracotomy (n = 147)	*p* value	VATS (n = 98)	Thoracotomy (n = 147)	Conversion (n = 23)	*p* value
Early mortality	1 (0.8)	1 (0.7)	1.000	1 (1.0)	1 (0.7)	0	0.869
**Postoperative event**							
Pneumonia	5 (4.1)	11 (7.5)	0.372	3 (3.1)	11 (7.5)	2 (8.7)	0.304
Atelectasis requiring BFS	1 (0.8)	5 (3.4)	0.316	1 (1.0)	5 (3.4)	0	0.350
Wound infection	1 (0.8)	2 (1.4)	0.982	1 (1.0)	2 (1.4)	0	0.841
Arrhythmia	2 (1.7)	7 (4.8)	0.287	1 (1.0)	7 (4.8)	1 (4.3)	0.271
Bleeding requiring surgery	1 (0.8)	1 (0.7)	1.000	1 (1.0)	1 (0.7)	0	0.869
Vocal cord palsy	0	5 (3.4)	0.111	0	5 (3.4)	0	0.123
Chylothorax	3 (2.5)	6 (4.1)	0.701	1 (1.0)	6 (4.1)	2 (8.7)	0.142

Data are presented as number (%). *BFS* bronchofibroscopy; video-assisted thoracoscopic surgery.

### Survival analysis

In the overall cohort before IPTW adjustment, 61 (50.4%) of the 121 patients in the VATS group and 81 (55.1%) of the 147 patients in the thoracotomy group had died at the end of the follow-up period; their 5-year overall survival (OS) rates were 49.6% and 52.6%, respectively. Recurrence events occurred in 47 and 56 patients in the VATS and thoracotomy groups, respectively; their 5-year recurrence-free survival (RFS) rates were 52.5% and 50.9%. The pattern of recurrence was not different between the two groups (Table S1).

**Figure 2:**
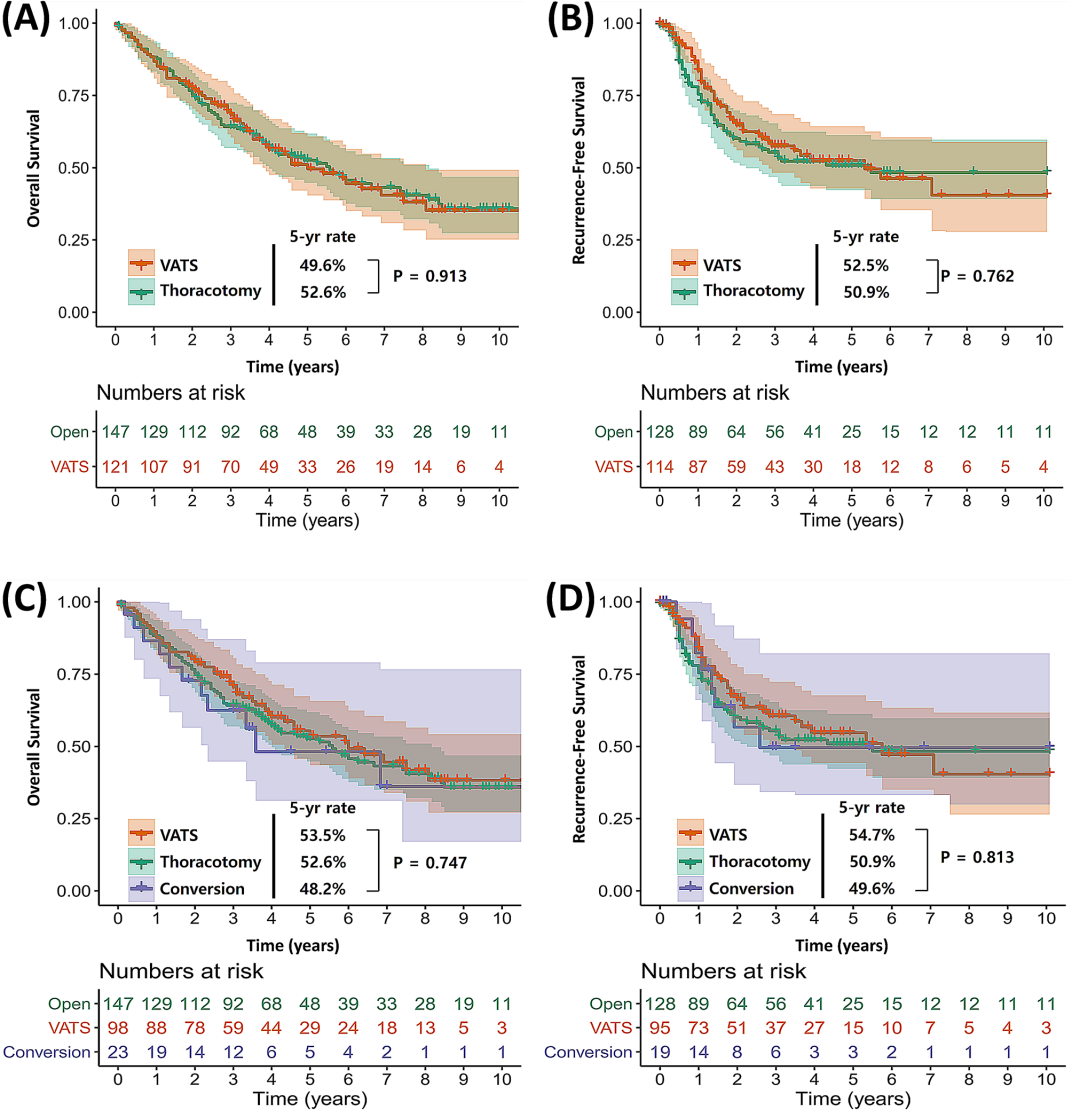
Overall survival (**A**) and recurrence-free survival (**B**) of patients with clinical N2 disease following the surgical procedure (VATS vs. thoracotomy) in the entire cohort. Overall survival (**A**) and recurrence-free survival (**B**) of patients with clinical N2 disease following the surgical procedure (VATS vs. thoracotomy vs. thoracotomy conversion) in the entire cohort.

**Figure 3:**
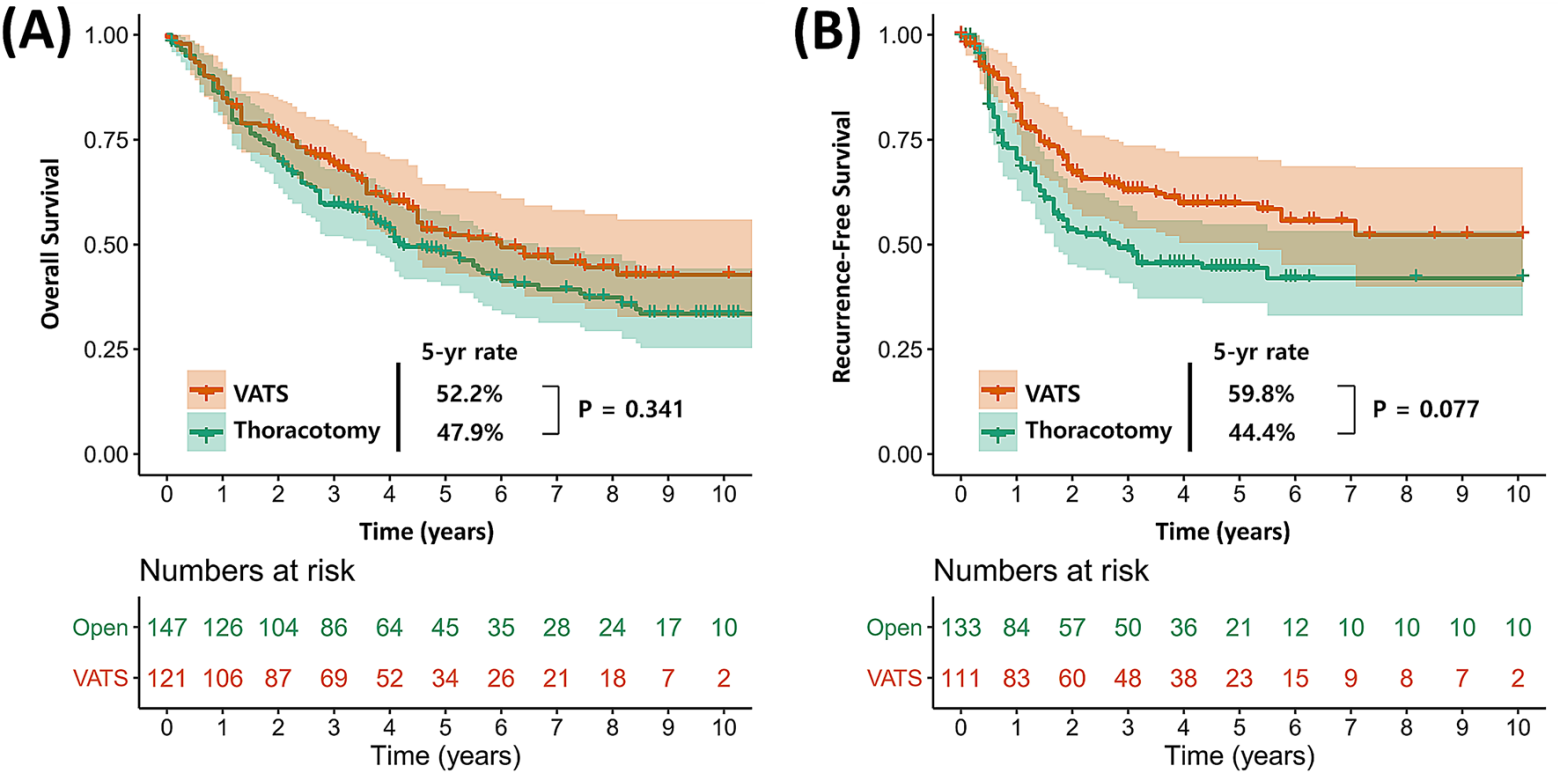
Overall survival (**A**) and recurrence-free survival (**B**) of patients with clinical N2 disease following the surgical procedure (VATS vs. thoracotomy) after IPTW adjustment.

The Kaplan–Meier survival curves, according to the surgical approach, are plotted in Fig. 2. The VATS group had similar survival outcomes with the thoracotomy group in terms of both OS (*p* = 0.913) and RFS (*p* = 0.762) (Fig. 2A, B). After including patients who underwent thoracotomy conversion during VATS in a conversion subgroup, there were no significantly different survival outcomes between the VATS, planned thoracotomy, and conversion groups (OS: *p* = 0.747; RFS: *p* = 0.813) (Fig. 2C, D). After IPTW adjustment, OS curves were still similar between the VATS and thoracotomy groups (*p* = 0.127) (Fig. 3A). However, the VATS group had a better RFS rate than the thoracotomy group, albeit with marginal significance (*p* = 0.069) (Fig. 3B). On univariable Cox analysis after IPTW adjustment, the VATS approach was not a significant prognostic factor for OS (hazard ratio [HR] (95% confidence interval [CI]) = 0.86 (0.57–1.29), *p* = 0.461), whereas it was for RFS among patients with cN2 disease (HR (95% CI) = 0.64 (0.43–0.96), *p* = 0.030) (Table S2). It was still an independent prognostic factor for RFS on multivariable analysis in the IPTW-adjusted cohort (HR (95% CI) = 0.63 (0.42–0.95), *p* = 0.017) (Table S3).

## Discussion

We evaluated the perioperative and long-term outcomes of NSCLC patients who received VATS versus open lobectomy for the treatment of clinical stage IIIA cancer due to suspected N2 node metastasis. The VATS approach was associated with a shorter length of hospitalization and time to initial postoperative chemotherapy compared with the open thoracotomy approach. Furthermore, the rate of complete resection, mean number of dissected LNs, and rate of postoperative complications were similar between the two groups. Importantly, on both IPTW-unadjusted and IPTW-adjusted analysis, the VATS group showed no significant observed difference in 5-year OS and a marginally better RFS, compared with the thoracotomy group. According to multivariable Cox analysis, the VATS approach was not a significant prognostic factor of OS and RFS among patients with cN2 disease.

In our institution, upfront surgical resection followed by adjuvant therapy has been prospectively performed for patients with resectable N2 disease for 20 years^15^. Initially, open thoracotomy was usually performed for these patients. Later, the accumulation of clinical experience and advances in instrument development facilitated the application of VATS for patients with cN2 disease, and in 2016, the rate of VATS implementation for cN2 disease increased to 73.3% (22/30). However, compared with open thoracotomy, VATS lobectomy tended to be performed less frequently for male patients as well as those with a smoking history, squamous cell carcinoma, large tumors, and centrally located tumors (Table 1). These clinical findings are linked to each other solely by the central tumor factor. Thus, we concluded that VATS lobectomy for patients with cN2 disease was preferred for patients with peripherally located tumors.

One potential concern about the VATS approach is the possibility of insufficient LN dissection owing to the narrow visual field and limited range of instrumental movement. Previous studies have reported that the number of harvested LNs in the VATS group was fewer than that in the thoracotomy group^16,17^. However, recently, the number and quality of LNs harvested via VATS lobectomy were found to be similar to those harvested via open lobectomy^18,19^. In our study, there were no significant differences in the mean number of harvested LNs (31.9 vs. 29.4, *p* = 0.100) or positive LNs (4.6 vs. 4.7, *p* = 0.980) in patients with cN2 disease (Table 2).

The other concern is increased morbidity and mortality among patients who undergo thoracotomy conversion from VATS. According to previous reports, unexpected thoracotomy conversion, with rates ranging from 5 to 23%, has been associated with greater perioperative morbidity compared with successful VATS completion^20,21^. In contrast, similar postoperative complications were observed between converted and planned thoracotomy patients^20,21^. A recent study based on the National Cancer Database also reported similar complication rates and long-term survival between patients with planned and converted thoracotomy for clinical N1 disease^9^. In our study, the rate of thoracotomy conversion was 19% (23/121), and the rate of emergency cases due to vascular injury was 13.0% (3/23). Also, the VATS, thoracotomy, and conversion groups had similar perioperative morbidity, early mortality (Table 3), and long-term mortality rates (Fig. 2).

Although there were no significant differences in OS and RFS between the two groups, it is worth mentioning that the differences became larger after IPTW adjustment (Figs. 2 and 3). The 5-year RFS rates of the VATS and thoracotomy groups were 52.5% and 50.9% (*p* = 0.762), respectively, whereas they were 60.5% and 44.6% (*p* = 0.069) on IPTW-adjusted analysis. Additionally, the VATS approach was associated with better RFS on multivariable Cox analysis (HR (95% CI) = 0.63 (0.42–0.95), *p* = 0.026). This phenomenon can be explained by comparing the differences in the pathologic findings before and after IPTW adjustment. As described in Table 2, the rate of pathologic N2 status was slightly higher in the VATS group than in the thoracotomy group (68.6% vs. 60.5%) but became similar after IPTW adjustment (67.8% vs. 66.0%). Also, the rate of adjuvant chemotherapy was similar between the two groups (53.7% vs. 52.4%) but became higher in the VATS group (54.5% vs. 46.9%) after IPTW adjustment. Therefore, according to our IPTW-adjusted analysis, the VATS approach was associated with better RFS than the thoracotomy approach among identical patients with cN2 disease, owing to the enhanced compliance of adjuvant chemotherapy.

This study had notable limitations. First, selection bias is inherent in a retrospective study from a single institution, although the data in this study were gathered prospectively. Second, as in most studies comparing two surgical approaches, there might be another selection bias stemming from the surgeons’ preferences. We minimized the bias as much as possible by using a PS-based IPTW method. Third, the proportion of patients who received adjuvant chemotherapy, and the frequency of recurrence events were relatively low. Because our hospital is a tertiary referral center, some patients were followed-up at local hospitals, and we could not assess their postoperative information. However, given that similar clinical characteristics, rates of recurrence, and patterns of recurrence were shown between the two groups after IPTW, these unknown data cannot change our conclusions.

In conclusion, VATS may be a safe and feasible approach for patients with resectable cN2 disease and a peripherally located tumors, associated with a shorter duration of hospitalization and time to adjuvant chemotherapy initiation, as well as comparable perioperative and long-term survival outcomes, relative to open thoracotomy. When it is performed by surgeons who have adequate experience and who carry out careful surgical planning, VATS lobectomy can be an appropriate option for selected patients with clinical N2 disease without compromising oncologic efficacy.

## Methods

### Patients

All clinical records of patients who underwent surgery for NSCLC between January 2007 and December 2016 were retrospectively reviewed from a prospectively gathered lung cancer database at Asan Medical Center in Seoul, South Korea. During the study period, 6,145 patients with NSCLC underwent curative-intent surgery for primary lung cancer, and 681 (11.2%) of them were diagnosed with stage IIIA cN2 disease.

The exclusion criteria were: (1) concomitant malignancies (n = 23), (2) administration of neoadjuvant therapy (n = 109), (3) pneumonectomy or sublobar resection (wedge resection and segmentectomy) (n = 50), (4) distant metastasis (n = 95), and (5) history of lung cancer treatment in the past 2 years (n = 13). Because the VATS approach is not feasible for patients with advanced T stage, patients with clinical T3 and T4 disease were also excluded (n = 123) (Fig. 1). According to intent-to-treat analysis^22^, patients converted to thoracotomy were included in the VATS group. This study was performed in accordance with relevant guidelines and was approved by the Asan Medical Center Institutional Review Board. Informed consent was obtained from all participants in studies that involve human subjects.

### Preoperative and postoperative management

The preoperative staging workup consisted of medical history, physical examination, complete blood counts, blood chemistry with electrolytes, chest X-ray, computed tomography (CT) of the chest and upper abdomen, biopsy, bronchoscopy, pulmonary function testing, radionuclide bone scanning, and 18F-fluorodeoxyglucose (FDG) positron emission tomography (PET). Brain CT or magnetic resonance imaging with contrast was conducted routinely as part of the staging workup^23^.

The following definition of cN2 disease was used: enlarged LNs (defined as ≥ 10 mm on the largest short axis) on chest CT or PET-positive LNs in an N2 position, according to the Mountain–Dresler modification of the American Thoracic Society (MD-ATS) LN map^24^. LNs were considered positive on PET if FDG uptake was higher than background uptake in the mediastinal blood pool. In cases of borderline tumor size or metabolic uptake of mediastinal LNs on CT or PET, biopsies of the involved mediastinal LNs were conducted, with mediastinoscopy, endobronchial ultrasound, or endoscopic ultrasound. Treatment approaches for cN2 disease were determined by a multidisciplinary team, including medical oncologists, radiologists, and thoracic surgeons. Upfront surgery for cN2 disease was generally considered when (1) the primary tumor was resectable without pneumonectomy, (2) mediastinal LN metastasis was confined within a single zone (upper zone for LNs at stations 2–4, aortopulmonary zone for stations 5 and 6, subcarinal zone for station 7, and lower zone for stations 8 and 9)^25^, (3) the involved LN was distinct from surrounding tissues and less than 3.0 cm in diameter, and (4) there was no sign of extra-nodal tumor invasion (the presence of full-thickness LN capsular invasion or extension of tumor cells beyond the LN capsule) on CT or PET.

Follow-up information on all patients was obtained through clinic follow-up notes every 3 months for the first 2 years, every 6 months for the next 3 years, and annually thereafter. Chest CT scans were performed at the time of clinical visits or at any time when disease recurrence was suspected. Treatment modalities and chemotherapeutic regimens in relapsed cases were determined at the discretion of the attending physician^23^.

### Operative technique

The decision to perform VATS or open thoracotomy for cN2 disease was made by each of the four surgeons individually. However, the VATS approach was primarily considered for resectable cN2 disease, except when centrally located tumors or calcified LNs were very close to hilar structures on preoperative images. Under general anesthesia using one-lung ventilation by double-lumen endotracheal intubation, VATS was performed in the full lateral position with a 30° angled thoracoscope and the usual three-port technique. The incision sites varied slightly by surgeon but can be broadly classified into two methods. The first method involved making an approximately 4 cm utility incision in the mid-axillary line through the fourth or fifth intercostal space (ICS), a 1.2 cm camera port in the anterior axillary line through the seventh ICS, and a 1.5 cm incision in the posterior axillary line at the same level of the camera port. The second method consisted of a camera port at the mid-axillary line at the eighth ICS, an approximately 4 cm working port at the anterior axillary line at the fifth ICS, and another instrument port at the posterior axillary line at the sixth ICS. To decrease operative bleeding and avoid thermal injury along the nerve trajectory, bipolar or ultrasonic energy devices were routinely used for the VATS approach.

Operative procedures for cN2 disease included resection of the primary lung cancer and systemic LN dissection of the ipsilateral hilum and the mediastinum. Mediastinal LN dissection consisted of en bloc resection of all nodes at stations 2R, 4R, 7, 8, 9, 10R, and 11R for right-sided tumors and nodes at stations 5, 6, 7, 9, 10L, and 11L (selectively with 4L for the upper lobe, 8 and 9 for the lower lobe) for left-sided tumors, regardless of surgical approach. LN dissection with the adipose connective tissue of the related anatomic regions was performed, as determined intraoperatively by the surgeon. All LNs, either dissected intraoperatively or harvested later from specimens, were examined pathologically and classified based on anatomic location by the numbering system described in the MD-ATS^24^. Histopathological staging was performed according to the American Joint Committee on Cancer (AJCC) 8th edition^26^.

### Definitions

Considering that, in a predictive model for postoperative mortality, the number of comorbid diseases per patient is superior to individual comorbidities, such as hypertension, diabetes mellitus, chronic bronchitis, coronary artery disease, cardiac arrhythmia, congestive heart failure, liver cirrhosis, cerebral vascular events, renal insufficiency, history of malignant disease, and prior thoracic surgery^27^, we considered this to be a categorical variable. Conversion to thoracotomy was defined as operations that began with thoracoscopic dissection and concluded as a rib-spreading thoracotomy. Centrally located tumors were defined as those in contact with the lobar pulmonary artery, vein, or bronchus, or their first segmental branches on CT^28^.

N1 and N2 status were subdivided depending on the number of involved LN stations (single vs. multiple): N1 was divided into N1 single (N1a) and N1 multiple (N1b), and N2 was also divided into N2 single (N2a) and N2 multiple (N2b). The presence of skip metastasis was also taken into consideration: N2a was divided into N2 single with skip (no N1 involvement, N2a1) and N2 single without skip (N1 involvement as well, N2a2) (Table S4)^29^.

OS was calculated as the time from the date of resection to the date of death using the records from the domestic National Security Death Index Database. RFS was defined as the time from the date of operation to the date of recurrence, while patients without recurrence were censored at the latest time point at which they were known to be recurrence-free. Patients with incomplete resection were excluded from recurrence-related outcomes, as recurrence connotes previous potential eradication of all disease.

### Statistical analysis

Student’s t tests or Mann–Whitney tests, depending on the normality of distribution, and chi-square or Fisher’s exact tests were applied to compare continuous and categorical variables, respectively. The normality of the distributions of individual variables was checked using the Shapiro–Wilk test. For comparisons among the three groups, we used a parametric test (ANOVA) for continuous variables and the chi-square test for categorical variables. Survival curves reflecting OS and RFS were plotted using the Kaplan–Meier method and were compared univariately using the log-rank test. Cox proportional hazard models were used to identify the predictors of mortality and recurrence. After excluding the correlated variables, independent variables with *p* values ≤ 0.05 from the univariate analysis were used for the initial multivariate Cox analysis. The final multivariable model was selected using forward stepwise selection (*p* value ≤ 0.10 for entering the model and *p* ≤ 0.05 for staying in the model). The proportional hazards assumption for the Cox regression models was assessed with Schoenfeld residuals.

The differences in the baseline patient characteristics between the VATS and thoracotomy groups were adjusted with a propensity score (PS)-based IPTW technique^30^. The PS, which indicated the probability of using VATS, was estimated by multiple logistic regression analysis using the 11 baseline variables (Table 1). The weights for patients were calculated using the formula 1/PS for VATS and 1/(1 − PS) for thoracotomy. To evaluate the balance between the two groups, standardized mean differences were used^30,31^. The impact of VATS on postoperative and long-term clinical outcomes was evaluated using weighted logistic regression and weighted Cox proportional hazards modeling, respectively. The proportional hazards assumption in the Cox model was evaluated using Schoenfeld residuals. The adjusted Kaplan–Meier curves were formulated to delineate the OS and RFS^32^.

All statistical calculations were performed using R version 3.4.2 (The R Foundation for Statistical Computing, Vienna, Austria) using the “Survival”, “MatchIt”, “dplyr”, “smd", “ggplot2”, “GGally”, “survminer”, “Zelig”, “twang” and “rms” packages. A P value less than 0.05 was considered statistically significant.

## Supplementary information

Supplementary Table S1.

Supplementary Table S2.

Supplementary Table S3.

Supplementary Table S4.
