# Supplementary material for: Video-assisted thoracoscopic lobectomy is feasible for selected patients with clinical N2 non-small cell lung cancer
Source: Sci Rep. 2020 Sep 16;10:15217. doi: 10.1038/s41598-020-72272-4 (PMC7495470; doi:10.1038/s41598-020-72272-4)
Supplement: Supplementary file 1 — Supplementary Table S1. [file 41598_2020_72272_MOESM1_ESM.docx]

Table S1. Recurrence pattern in patients with clinical N2 disease

| Variables | VATS  (n=114) | Thoracotomy  (n=128) |
| --- | --- | --- |
| **Total numbers** | 47 | 56 |
| **Loco-regional recurrence** | 9 | 17 |
| Bronchial stump | 1 | 3 |
| Ipsilateral lung | 0 | 2 |
| Ipsilateral lymph node | 2 | 7 |
| Others | 5 | 4 |
| **Distant** | 40 | 42 |
| Brain | 14 | 7 |
| Bone | 6 | 16 |
| Contralateral lung | 19 | 13 |
| Contralateral lymph node | 7 | 8 |
| Others | 9 | 12 |
| **Mixed** | 4 | 8 |
