# Supplementary material for: Video-assisted thoracoscopic lobectomy is feasible for selected patients with clinical N2 non-small cell lung cancer
Source: Sci Rep. 2020 Sep 16;10:15217. doi: 10.1038/s41598-020-72272-4 (PMC7495470; doi:10.1038/s41598-020-72272-4)
Supplement: Supplementary file 2 — Supplementary Table S2. [file 41598_2020_72272_MOESM2_ESM.docx]

Table S2. Univariable analyses of prognostic factors of NSCLC patients with clinical N2 disease after IPTW-adjustment.

| Variables | Overall survival | | |  | Recurrence-free survival | | |
| --- | --- | --- | --- | --- | --- | --- | --- |
|  | HR | 95% CI | P value |  | HR | 95% CI | P value |
| **Age, year** | 1.02 | 0.99-1.04 | 0.086 |  | 0.97 | 0.95-0.99 | 0.009 |
| **Sex, male** | 0.94 | 0.62-1.42 | 0.767 |  | 0.52 | 0.34-0.80 | 0.003 |
| **Presence of smoking history** | 0.92 | 0.61-1.39 | 0.689 |  | 0.62 | 0.40-0.97 | 0.037 |
| **Comorbidities per patient, n** |  |  |  |  |  |  |  |
| 0 | 1 | ref. |  |  | 1 | ref. |  |
| 1 | 0.80 | 0.51-1.25 | 0.328 |  | 0.61 | 0.38-0.97 | 0.038 |
| 2 | 1.26 | 0.71-2.23 | 0.440 |  | 0.99 | 0.53-1.85 | 0.967 |
| ≥ 3 | 2.47 | 1.21-5.03 | 0.013 |  | 0.79 | 0.35-1.78 | 0.568 |
| **Pulmonary function** |  |  |  |  |  |  |  |
| FEV1 | 0.99 | 0.97-1.00 | 0.069 |  | 1.00 | 0.98-1.02 | 0.957 |
| DLCO | 0.99 | 0.98-1.00 | 0.143 |  | 1.00 | 0.99-1.02 | 0.854 |
| **Tumor location** |  |  |  |  |  |  |  |
| Peripheral | 1 | ref. |  |  | 1 | ref. |  |
| Central | 0.83 | 0.56-1.23 | 0.358 |  | 0.78 | 0.51-1.21 | 0.267 |
| **Histologic structure** |  |  |  |  |  |  |  |
| ADC* | 1 | ref. |  |  | 1 | ref. |  |
| SqCC* | 0.71 | 0.47-1.07 | 0.102 |  | 0.32 | 0.20-0.52 | <0.001 |
| Others | 0.69 | 0.29-1.64 | 0.402 |  | 0.26 | 0.09-0.70 | 0.008 |
| **Surgical approach** |  |  |  |  |  |  |  |
| Thoracotomy | 1 | ref. |  |  | 1 | ref. |  |
| VATS | 0.86 | 0.57-1.29 | 0.461 |  | 0.64 | 0.43-0.96 | 0.030 |
| **Resection margins** |  |  |  |  |  |  |  |
| R0 | 1 | ref. |  |  | - | - | - |
| R1 | 1.39 | 0.81-2.40 | 0.234 |  | - | - | - |
| **Tumor size, mm** | 0.99 | 0.97-1.00 | 0.093 |  | 0.99 | 0.97-1.00 | 0.123 |
| **Pathologic T factor** |  |  |  |  |  |  |  |
| T1 | 1 | ref. |  |  | 1 | ref. |  |
| T2 | 1.41 | 0.85-2.35 | 0.181 |  | 1.05 | 0.65-1.71 | 0.835 |
| T3 | 0.51 | 0.23-1.10 | 0.082 |  | 0.48 | 0.21-1.10 | 0.083 |
| **Pathologic N factor** |  |  |  |  |  |  |  |
| N0 | 1 | ref. |  |  | 1 | ref. |  |
| N1 | 3.20 | 1.52-6.72 | 0.002 |  | 2.71 | 1.01-7.34 | 0.049 |
| N2 | 4.00 | 2.37-6.76 | <0.001 |  | 8.62 | 4.48-16.57 | <0.001 |
| **Adjuvant chemotherapy** | 0.89 | 0.60-1.33 | 0.571 |  | 1.50 | 0.94-2.40 | 0.087 |
| **Adjuvant radiotherapy** | 1.33 | 0.90-1.96 | 0.157 |  | 1.77 | 1.13-2.77 | 0.012 |

HR: hazard ratio; CI: confidence interval; FEV1, forced expiratory volume during the first second; DLCO, diffusing capacity of carbon monoxide; ADC, adenocarcinoma; SqCC, squamous cell carcinoma; VATS, video-assisted thoracoscopic surgery; R0, completely resected tumor; R1, microscopic residual tumor.
