# Supplementary material for: Video-assisted thoracoscopic lobectomy is feasible for selected patients with clinical N2 non-small cell lung cancer
Source: Sci Rep. 2020 Sep 16;10:15217. doi: 10.1038/s41598-020-72272-4 (PMC7495470; doi:10.1038/s41598-020-72272-4)
Supplement: Supplementary file 3 — Supplementary Table S3. [file 41598_2020_72272_MOESM3_ESM.docx]

Table S3. Multivariable analyses of prognostic factors of NSCLC patients with clinical N2 disease after IPTW-adjustment.

| Variables | Overall survival | | |  | Recurrence-free survival | | |
| --- | --- | --- | --- | --- | --- | --- | --- |
|  | HR | 95% CI | P value |  | HR | 95% CI | P value |
| **Age, year** | 1.03 | 1.01-1.06 | 0.016 |  |  |  |  |
| **Comorbidities per patient, n** |  |  |  |  |  |  |  |
| 0 | 1 | ref. |  |  |  |  |  |
| 1 | 0.61 | 0.40-0.94 | 0.024 |  |  |  |  |
| 2 | 1.21 | 0.75-1.95 | 0.430 |  |  |  |  |
| ≥ 3 | 1.43 | 0.65-3.16 | 0.379 |  |  |  |  |
| **Histologic structure** |  |  |  |  |  |  |  |
| ADC* |  |  |  |  | 1 | ref. |  |
| SqCC* |  |  |  |  | 0.55 | 0.34-0.90 | 0.017 |
| Others |  |  |  |  | 0.38 | 0.12-1.18 | 0.095 |
| **Surgical approach** |  |  |  |  |  |  |  |
| Thoracotomy |  |  |  |  | 1 | ref. |  |
| VATS |  |  |  |  | 0.63 | 0.42-0.95 | 0.026 |
| **Pathologic N factor** |  |  |  |  |  |  |  |
| N0 | 1 | ref. |  |  | 1 | ref. |  |
| N1 | 6.16 | 2.82-13.46 | <0.001 |  | 3.27 | 1.27-8.40 | 0.014 |
| N2 | 7.83 | 4.44-13.83 | <0.001 |  | 9.10 | 3.80-21.82 | <0.001 |
| **Adjuvant chemotherapy** | 0.53 | 0.35-0.80 | 0.003 |  | 0.64 | 0.35-0.85 | 0.007 |
| **Adjuvant radiotherapy** |  |  |  |  | 0.57 | 0.36-0.90 | 0.016 |

HR: hazard ratio; CI: confidence interval; FEV1, forced expiratory volume during the first second; DLco, diffusing capacity of carbon monoxide; ADC, adenocarcinoma; SqCC, squamous cell carcinoma; VATS, video-assisted thoracoscopic surgery
