# Supplementary material for: Video-assisted thoracoscopic lobectomy is feasible for selected patients with clinical N2 non-small cell lung cancer
Source: Sci Rep. 2020 Sep 16;10:15217. doi: 10.1038/s41598-020-72272-4 (PMC7495470; doi:10.1038/s41598-020-72272-4)
Supplement: Supplementary file 4 — Supplementary Table S4. [file 41598_2020_72272_MOESM4_ESM.docx]

Supplementary Table S4. Definition of the N descriptors proposed by the International Association for the Study of Lung Cancer (IASLC)

| Category | Description |
| --- | --- |
| N0 | No regional lymph node involvement |
| N1 | Peribronchial, interlobar, or perihilar lymph nodes involved |
| N1a | Single-station N1 involvement |
| N1b | Multiple-station N1 involvement |
| N2 | Ipsilateral mediastinal nodes involved |
| N2a1 | Single-station N2 without N1 involvement (skip metastasis) |
| N2a2 | Single-station N2 with N1 involvement |
| N2b | Multiple-station N2 involvement |
| N3 | contralateral mediastinal or supraclavicular nodes involved |
